# Genome-Wide Characterization of a Recombinant BoAHV-1 ΔgE/β-gal Vaccine Strain Reveals an Unintended Multi-Gene Deletion

**DOI:** 10.3390/microorganisms14102396

**Published:** 2026-10-20

**Authors:** Silvina Soledad Maidana, Carola Ferreccio, Ana Marandino, Yanina Panzera, Ruben Pérez, Sonia Alejandra Romera

**Affiliations:** 1 Institute of Virology and Technological Innovations, Dr. Nicolás Repetto and De los Reseros, IVIT (INTA-CONICET), Hurlingham 1686, Argentina; 2 Evolutionary Genetics Section, Faculty of Sciences, Institute of Biology, University of the Republic, Montevideo 11400, Uruguayypanzera@fcien.edu.uy (Y.P.);; 3 Faculty of Agricultural and Veterinary Sciences, Veterinary Research Institute, University of the Salvador, Buenos Aires 1426, Argentina

**Keywords:** bovine alphaherpesvirus 1, recombinant vaccine, genome-wide, structural variation, gene deletion

## Abstract

Bovine alphaherpesvirus 1 (BoAHV-1) is a major cattle pathogen, and glycoprotein E (gE)-negative marker vaccines are widely used for disease control and to differentiate infected from vaccinated animals. The genomic architecture of this previously developed recombinant strain beyond the intended gE/lacZ locus had not been characterized at the genome-wide level. Here, we performed whole-genome sequencing of the parental Los Angeles (LA) strain and two passages of the recombinant BoAHV-1ΔgEβgal strain. In addition, plaque area was descriptively assessed in primary fetal bovine kidney cells using measurements from a single experimental run. In addition to the expected replacement of the US8/gE locus with a β-galactosidase cassette, we identified a previously undescribed 3704 nt deletion in the US region. This deletion removed the complete US1.67, US2, and US3 coding sequences, as well as the 5′ portion of US4/gG. PCR assays and Sanger sequencing of the amplicon spanning the breakpoint precisely mapped the junction. Comparative annotation showed that the parental genome contained 74 viral CDSs, whereas both vaccine passage genomes contained 69. In the plaque assay, the mean plaque area of p11 was 68.8% lower than that of the parental LA strain. These findings document an unintended structural alteration in a previously evaluated recombinant vaccine strain and illustrate how genome-wide sequencing can complement targeted molecular characterization by identifying changes outside the intended engineered locus.

## 1. Introduction

Bovine alphaherpesvirus 1 (BoAHV-1; species *Varicellovirus bovinealpha1*) is the etiological agent of infectious bovine rhinotracheitis (IBR), a disease with major economic impact on cattle production worldwide [1]. A key feature of BoAHV-1 infection is its ability to establish lifelong latency in sensory ganglia, with periodic reactivation and viral shedding that complicate disease control and eradication efforts [2,3].

The BoAHV-1 genome is a linear, double-stranded DNA molecule with the typical class D herpesvirus genome organization, consisting of a unique long (UL) region and a unique short (US) region. The US region is flanked by two inverted repeat sequences, the internal repeat short (IRS) and terminal repeat short (TRS) regions [1]. This architecture is biologically relevant because these repeated sequences can promote homologous recombination and mediate inversion of the US segment, thereby generating alternative genomic isomers [4].

Vaccination remains the primary strategy for controlling IBR. Among the available approaches, gene-deleted marker vaccines, particularly those lacking glycoprotein E (gE), are widely used for their safety and to support disease surveillance programs [4,5,6]. For the previously developed BoAHV-1ΔgEβgal strain, the genomic architecture outside the intended gE/lacZ modification had not been characterized at genome-wide resolution. Spontaneous genomic rearrangements, including deletions affecting virulence-associated genes, have been reported in BoAHV-1 and other herpesviruses [7], illustrating the potential for additional genomic changes outside the intended engineered locus.

Recent advances in attenuation strategies have focused on deleting multiple virulence-associated genes to enhance vaccine safety. Experimental studies have shown that simultaneously disrupting genes involved in viral replication, cell-to-cell spread, and immune modulation can yield highly attenuated phenotypes while preserving immunogenicity [8,9]. In this context, the US region is of particular interest because it encodes several proteins involved in intracellular trafficking, cytoskeletal regulation, cell-to-cell dissemination, and host–virus interactions, including glycoproteins and tegument-associated proteins [5,10,11,12].

Our group previously developed a recombinant BoAHV-1ΔgEβgal strain that demonstrated high safety and efficacy in vivo, with reduced viral excretion and no evidence of horizontal or vertical transmission [13]. In the present study, we retrospectively characterized the genomic architecture of this previously evaluated recombinant vaccine strain using genome-wide sequencing. The objectives were to identify genomic changes beyond the intended gE/lacZ modification and to descriptively characterize plaque area in primary fetal bovine kidney cells, without assuming a causal relationship between additional genomic alterations and the observed phenotype or in vivo attenuation.

## 2. Materials and Methods

### 2.1. Cells and Viruses

The BoAHV-1.1 Los Angeles (LA) parental strain (ATCC VR-188) and the recombinant BoAHV-1ΔgEβgal vaccine strain (p8 and p11 passages) were propagated in Madin–Darby bovine kidney (MDBK) cells and primary fetal bovine kidney (PFBK) cells. Passages p8 and p11 of the vaccine strain were produced to obtain sufficient volume for the vaccine formulation used in the experimental trial with cattle [13]. Cells were maintained in Eagle’s Minimum Essential Medium (MEM) supplemented with 10% fetal bovine serum (FBS), 100 µg/mL streptomycin, and 100 IU/mL penicillin at 37 °C in a humidified atmosphere containing 5% CO_2_.

Viral stocks were generated by infecting MDBK cell monolayers and titrated by endpoint dilution. Briefly, tenfold serial dilutions were inoculated in quadruplicate onto confluent cells in 96-well plates. After 72 h, cytopathic effect (CPE) was assessed and viral titers were calculated using the Reed–Muench method [14].

### 2.2. DNA Extraction, Sequencing, and Genome Analysis

Viral DNA was extracted using the QIAamp DNA Mini Kit (Qiagen, Hilden, Germany) according to the manufacturer’s instructions. Sequencing libraries were prepared using the Nextera XT DNA Library Preparation Kit (Illumina, San Diego, CA, USA) and purified with AMPure XP beads (Beckman Colter, Brea, CA, USA). All quantifications were performed using a Qubit DNA High-Sensitivity kit (Invitrogen, Carlsbad, CA, USA). Library quality and length were assessed on a Fragment Analyzer 5200 system (Agilent Technologies, Santa Clara, CA, USA) with the Standard Sensitivity NGS Analysis Kit (Agilent Technologies, Santa Clara, CA, USA). Whole-genome sequencing was performed on an Illumina MiniSeq (Illumina, San Diego, CA, USA) platform using the MiniSeq™ Mid Output Reagent Cartridge (300 cycles, paired-end reads). We performed adapter and quality trimming, as well as raw data filtering, with BBDuk (version 1.0). Clean reads were mapped to the BoAHV-1.1 LA reference genome (GenBank MF421714.1) using Minimap2 (version 2.29) [15] as implemented in Geneious Prime (version 2026.0.2; Biomatters Ltd., Auckland, New Zealand) [16]. Consensus positions with insufficient or conflicting read support were encoded using IUPAC ambiguity symbols. These positions were not interpreted as nucleotide substitutions, indels, or structural variants. When located within CDSs, unresolved nucleotides resulted in X residues in the predicted protein translations. Comparative genomic conclusions were restricted to confidently resolved sequence positions. To resolve the engineered locus, filtered reads from p8 and p11 were additionally mapped to recombinant-specific reference sequences in which the βgal-containing cassette was incorporated into the LA genomic background according to the original construct design and the corresponding vector sequence. Genome annotations were transferred from the reference genome and manually curated, with special attention to the gE locus and the US-region deletion. Sequence alignments of consensus sequences were performed using MAFFT (Geneious MAFFT plugin 1.5.0) [17].

### 2.3. Consensus-Level Variant Analysis

To identify additional small sequence variants, the 69 annotated viral CDSs shared by the parental LA, p8, and p11 genomes were aligned and compared pairwise. Positions with N or other IUPAC ambiguity codes in either sequence were excluded from variant calling. Unambiguous nucleotide substitutions were classified as synonymous or nonsynonymous based on their predicted codon effect. Small insertions and deletions were also assessed within homologous CDSs. The engineered US8/lacZ replacement and the 3704 nt multi-gene deletion were analyzed separately as structural variants and were not included in the small-variant counts.

### 2.4. Sanger Sequencing of the Unintended Deletion Boundaries

To precisely define the deletion boundaries identified in the BoAHV-1ΔgEβgal p11 vaccine strain by genome sequencing, the genomic region spanning the deletion junction was amplified and analyzed by the Sanger method. Primers were designed (Del-F1: CGCCTTGCCTCTCTATATCTTG and Del-R1: GCTGCCCACGTAGATGTC) to anneal upstream and downstream of the deleted region, generating a 258 bp amplicon only when the deletion was present. This amplicon spanned 137 bp upstream and 121 bp downstream of the deletion breakpoint. PCR products were purified and subjected to bidirectional Sanger sequencing. The resulting sequences were aligned with the parental BoAHV-1 LA genome to determine the exact deletion boundaries and confirm its size and location.

### 2.5. PCR Analysis

Conventional PCR assays were carried out using five pairs of primers targeting US2, US3, US8 (gE), UL44 (gC), and the β-galactosidase gene. Primer sequences and expected amplicon sizes are listed in Table 1.

PCR reactions were carried out in 12.5 μL volumes containing 5 ng of template DNA, 1× GoTaq Green Buffer (Promega, Madison, WI, USA), 2 mM MgCl_2_, 200 μM dNTPs, 100 ng of each primer, 6% dimethyl sulfoxide (DMSO), and 1–2 U of Taq DNA polymerase (Invitrogen). Cycling conditions were optimized based on the melting temperatures of each primer pair.

Amplification products were resolved by electrophoresis in 1–2% agarose gels in 1× TAE buffer and visualized under UV illumination.

### 2.6. Plaque Assay

Plaque assays were performed in primary fetal bovine kidney (PFBK) cells seeded in 12-well plates. Cells were infected with tenfold serial dilutions of the parental LA wild-type (WT) strain and passage p11 of the recombinant BoAHV-1ΔgEβgal strain. Passage p11 was selected because it corresponds to the recombinant stock used in the previously published cattle study [13]. After 2 h of adsorption at 37 °C, the inoculum was removed, cells were overlaid with semisolid medium containing carboxymethyl cellulose (CMC), and the cultures were incubated for 72 h. Cells were fixed with 4% paraformaldehyde and stained with crystal violet. The available dataset, derived from a single experimental run, comprised 16 individual plaque measurements for parental LA and 11 for p11. Individual plaques were therefore treated as subsamples rather than independent biological replicates, and no inferential statistical test was applied.

Plaques were imaged, and their surface areas were quantified using ImageJ (version 1.12). Descriptive statistics, including the mean plaque area and standard deviation, were calculated for each group. The relative reduction in mean plaque area for p11 was calculated as [1 − (mean p11 plaque area/mean parental LA plaque area)] × 100. Individual plaque-area measurements are provided in Supplementary Table S1. 

## 3. Results

### 3.1. Genome Characterization of BoAHV-1 Strains

Genome-wide sequencing was performed on the parental BoAHV-1.1 Los Angeles (LA) strain used to generate the recombinant ΔgEβgal virus (parental LA strain) and on subsequent pre-formulation passages of the recombinant BoAHV-1ΔgEβgal strain (p8 and p11). High-quality Illumina data were obtained for all samples, with mean read lengths ranging from 145 to 148 bp. The LA parental consensus genome comprised 134,674 nt and was reconstructed from 485,988 reads, with a mean genome coverage of 402.5× (GenBank accession number PZ943071) and 99.9% pairwise identity relative to the BoAHV-1.1 LA reference genome MF421714. The two vaccine passage genomes comprised 133,807 nt and 133,905 nt, respectively. The first (p8) vaccine genome was assembled from 1,227,610 reads, with a mean coverage of 1187.8× (GenBank accession number PZ943072). In contrast, the second (p11) was assembled from 90,292 reads, with a mean coverage of 83.8× (GenBank accession number PZ943073).

Sequence ambiguity was quantified for each assembled genome. The LA parental, P8, and P11 genomes contained 2077 (1.542%), 920 (0.688%), and 2895 (2.162%) undetermined nucleotides (N), respectively. Of these, 1740, 101, and 2300 positions occurred within annotated coding sequences (CDSs), corresponding to 1.521%, 0.090%, and 2.052% of the total CDS length of LA parental, p8, and p11, respectively. The remaining 337, 819, and 595 N positions occurred outside CDSs. Most coding ambiguities were concentrated in a limited number of genes, particularly the repeat-associated BICP4 regions in parental LA and P11, whereas p8 showed substantially fewer ambiguous positions within CDSs. Because N positions represent unresolved nucleotides, their effects cannot be reliably classified as synonymous or nonsynonymous; codons containing ambiguous nucleotides were therefore considered indeterminate and excluded from variant-effect analyses. A complete gene-by-gene distribution of ambiguous positions is provided in Supplementary Table S2.

Read coverage across the β-galactosidase cassette was continuous in both recombinant passages, with a mean coverage of approximately 1852× in p8 (range, 685–2756×) and 126× in p11 (range, 67–195×). No reads supporting an intact wild-type US8/gE configuration were detected in either recombinant dataset.

**Figure 1:**
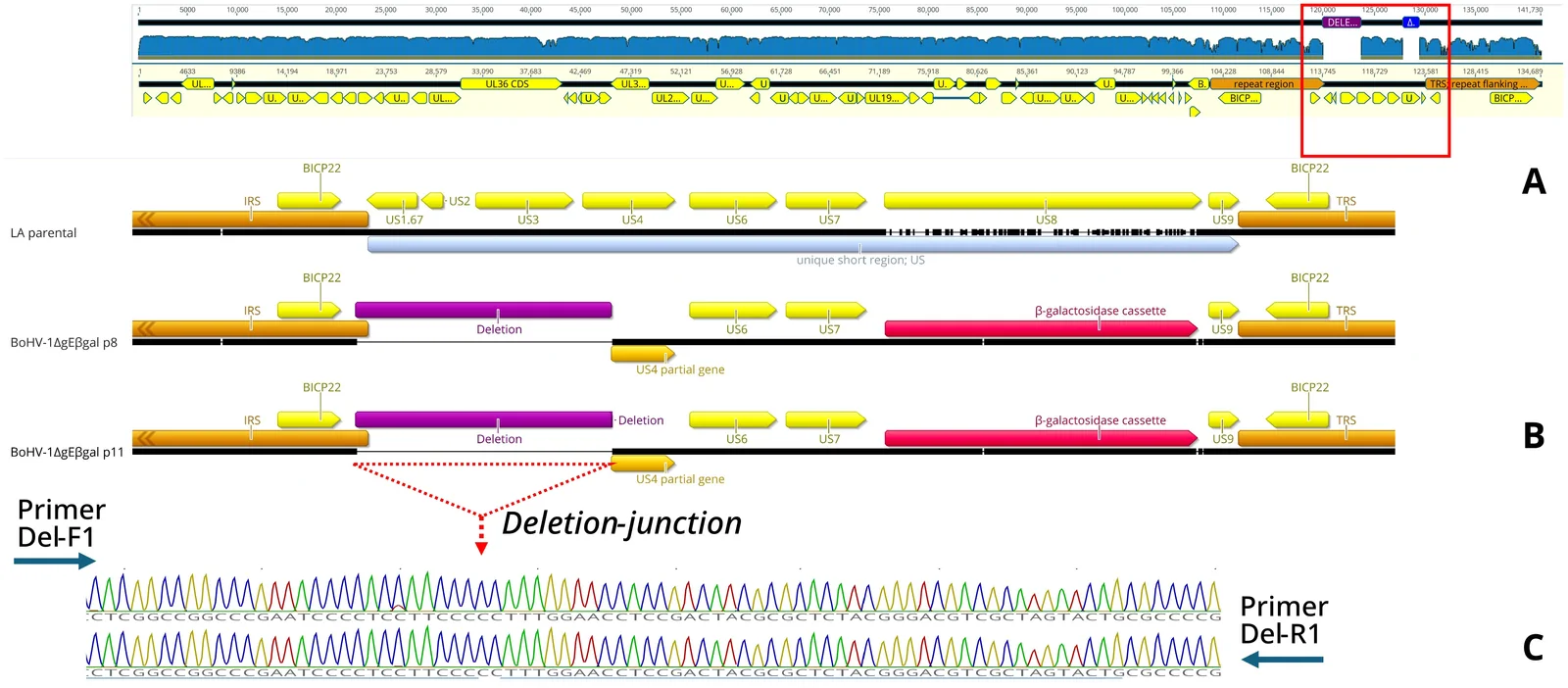
**Genome-wide, local, and Sanger-based confirmation of the deletion in the BoAHV-1ΔgEβgal vaccine strain.** (**A**) Genome-wide read-coverage profile of the recombinant vaccine strain mapped to the parental LA reference genome. The red box highlights the short genomic region containing the US-region locus corresponding to the engineered gE/lacZ modification and the additional deletion. (**B**) Comparative organization of the short genomic segment encompassing the US region and its flanking internal and terminal inverted repeats (IRS and TRS, respectively) in the LA parental strain and in the recombinant passages p8 and p11. The 3704 nt deletion, the retained partial US4 sequence, and the β-galactosidase cassette are indicated. US9 is located downstream of the engineered cassette and was retained in both recombinant passages. (**C**) Bidirectional Sanger sequencing of the p11 amplicon spanning the deletion junction. The chromatograms confirm that the sequences flanking the 3704 nt deleted interval join directly, independently validating the deletion site identified by genome-wide sequencing.

Comparative genome annotation showed that MF421714 and the LA parental genome contained 74 viral coding sequences (CDSs). In contrast, both vaccine passage genomes (p8 and p11) contained 69 viral CDSs. This reduction reflected the engineered replacement of the US8/gE locus with the engineered β-galactosidase (lacZ) cassette, as well as an unintended deletion in the US region. This unintended deletion spanned 3704 nt and encompassed the complete US1.67, US2, and US3 genes. The deletion also removes 419 nt from the 5′ end of the 1386 nt US4 CDS annotated in MF421714.1, including the annotated translation initiation codon, thereby disrupting the canonical full-length ORF (Figure 1).

The same 3704 nt deletion was detected in p8 and p11. Approximately 550 and 96 reads spanned the deletion junction in p8 and p11, respectively, and coverage on both sides of the breakpoint exceeded 100 reads. All junction-informative reads identified in both datasets supported the deleted configuration, whereas no reads supporting the corresponding wild-type junction were detected. These data indicate that the deleted genomic configuration was retained between p8 and p11, but do not establish when or how the deletion arose.

Apart from these structural modifications, the remaining viral CDSs were highly conserved between the LA parental and vaccine genomes. After excluding positions with ambiguous nucleotides, no unambiguous SNPs or small indels were identified in the 69 viral CDSs shared between the parental LA and p8 genomes. In p11, one unambiguous consensus-level nucleotide substitution relative to LA was identified in UL28 (CDS nucleotide 863, T→G), changing codon CTG to CGG and resulting in a predicted Leu288Arg substitution. No synonymous substitutions or additional small coding indels were detected. One additional nucleotide difference between p8 and p11 was observed in UL36 (CDS nucleotide 1265, T→G; Leu422Arg) (Supplementary Table S3). The engineered US8/lacZ replacement and the 3704 nt deletion were treated separately as structural variants.

### 3.2. Validation of Deletion Boundaries by PCR and Sanger Sequencing

The deletion junction was amplified as a 258 bp product from the BoAHV-1ΔgEβgal p11 vaccine strain and confirmed by bidirectional Sanger sequencing. The amplicon comprised 137 bp upstream and 121 bp downstream of the breakpoint, providing positive confirmation of the new junction created by the 3704 nt deletion.

Alignment against the parental BoAHV-1 LA genome precisely mapped the deletion boundaries and demonstrated a loss of 3704 nucleotides, encompassing the complete US1.67, US2, and US3 genes, as well as the 5′ portion of the US4 gene. Sanger sequencing confirmed the breakpoint, its size, and genomic location, consistent with the deletion detected by genome-wide sequencing (Figure 1).

### 3.3. Characterization of the Recombinant Vaccine Strain by PCR

**Figure 2:**
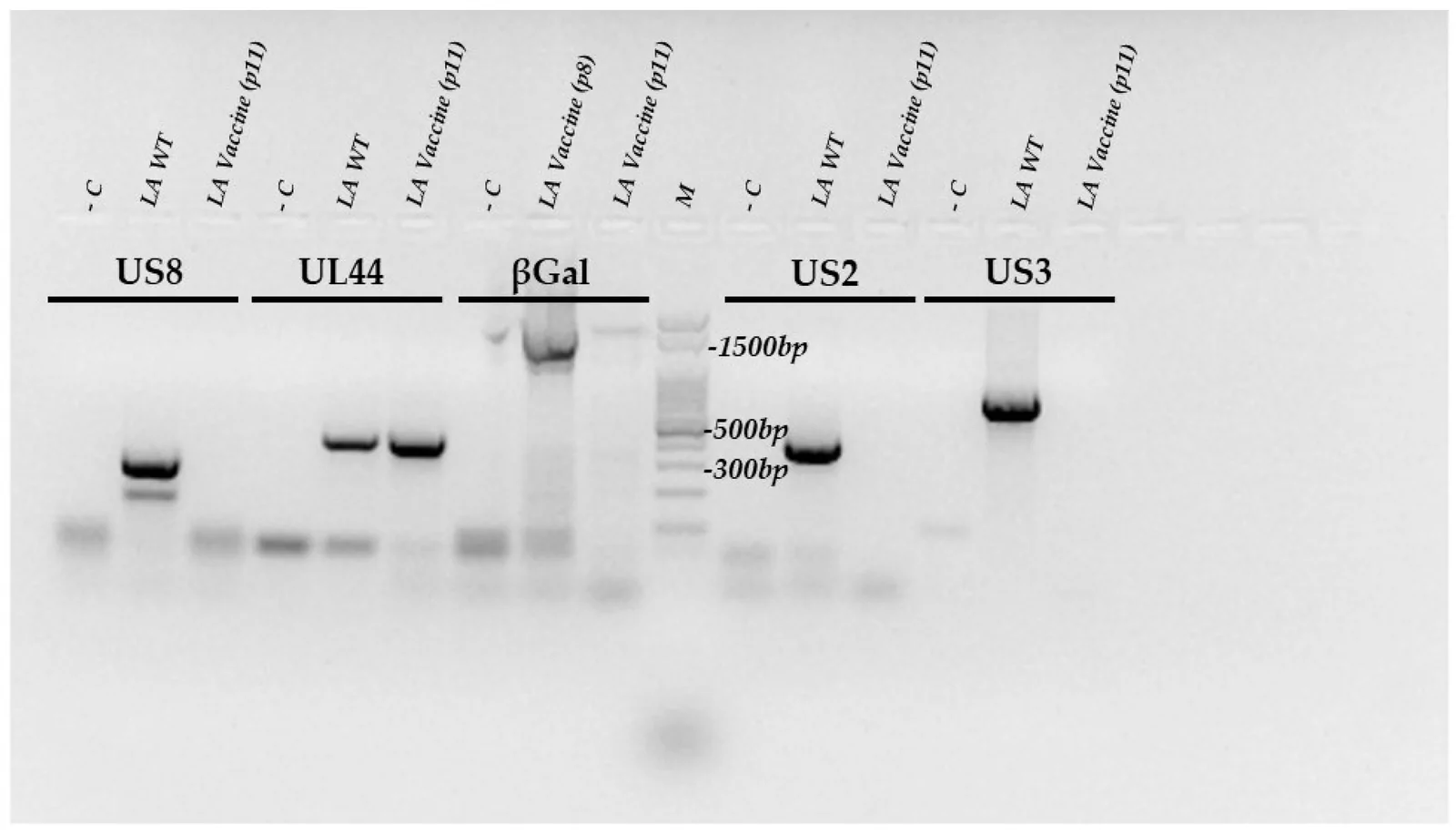
**Molecular characterization of the BoAHV-1ΔgEβgal vaccine strain.** Amplification of US2, US3, and US8 (gE) was detected in the parental LA WT strain but not in the BoAHV-1ΔgEβgal strain. The β-galactosidase insertion site was amplified from recombinant passages p8 and p11, whereas UL44/gC was amplified from the parental LA strain (LA WT) and from p11. The top panel shows the samples loaded into each lane and the five PCRs performed. Lanes 1–3 (US8/gE): negative control, LA WT, LA vaccine p11; lanes 4–6 (UL44/gC): negative control, LA WT, LA vaccine (p11); lanes 7–9 (lacZ/β-gal): negative control, LA vaccine (p8), LA vaccine (p11); lane 10: molecular-weight marker; lanes 11–13 (US2): negative control, LA WT, LA vaccine p11; lanes 14–16 (US3): negative control, LA WT, LA vaccine p11.

To independently validate the unintended deletion identified by sequencing, conventional PCR assays targeting selected genomic regions were performed. No amplification products were obtained for US2 or US3 from the BoAHV-1ΔgEβgal strain, consistent with both genes’ location within the unintended deletion. Both regions were readily amplified from the parental LA strain (Figure 2). As expected, US8/gE was not amplified from the recombinant strain because this locus had been replaced by the β-galactosidase cassette. Accordingly, amplification of the β-galactosidase insertion site and the UL44 (gC) gene, a CDS outside the deletion or recombinant regions, was observed in the vaccine-strain passages studied (Figure 2). These results were fully consistent with the genomic analysis and confirmed the loss of multiple genes within the US region.

### 3.4. Plaque-Area Measurements

**Figure 3:**
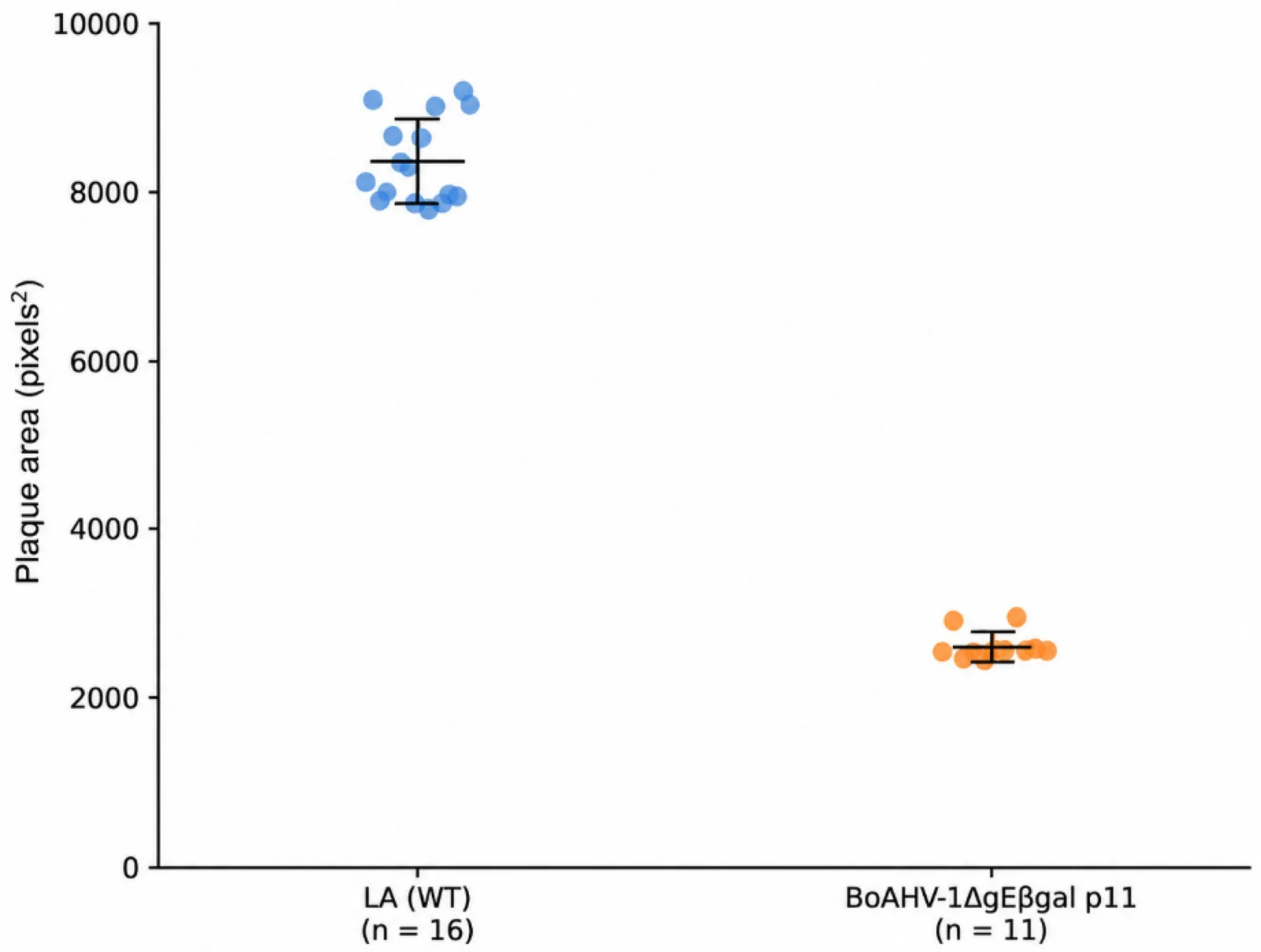
Plaque-area measurements of the parental BoAHV-1 LA and BoAHV-1ΔgEβgal p11 strains. Plaque areas were measured in primary fetal bovine kidney (PFBK) cells using ImageJ and are expressed in pixels^2^. Each point represents an individual plaque-area measurement (parental LA, *n* = 16; p11, *n* = 11). Horizontal lines indicate group means and vertical lines indicate standard deviations. Mean plaque areas were 8357.1 ± 504.5 pixels^2^ for LA and 2607.8 ± 164.8 pixels^2^ for p11, corresponding to a 68.8% lower mean plaque area for p11 under the experimental conditions tested.

The mean plaque area was 8357.1 ± 504.5 pixels^2^ for the parental LA strain and 2607.8 ± 164.8 pixels^2^ for p11, corresponding to a 68.8% lower mean plaque area for p11 in the available single-run dataset (Figure 3). Because individual plaques represented subsamples rather than independent biological replicates, these measurements were analyzed descriptively, and no inferential statistical comparison was conducted.

## 4. Discussion

The present study revealed an unintended multi-gene deletion in the US region of a recombinant BoAHV-1ΔgEβgal vaccine strain, extending beyond the originally engineered gE deletion.

Importantly, the deletion was independently validated by amplification and Sanger sequencing of the deletion junction. This approach allowed precise mapping of the breakpoint and confirmed the exact extent of the deleted region identified by next-generation sequencing. Short-read sequencing detected the same deleted genomic configuration in p8 and p11, indicating that it was retained between the two analyzed passages. PCR amplification and bidirectional Sanger sequencing of the deletion-junction amplicon independently confirmed the breakpoint identified from the short-read data (Figure 1).

Sequencing depth differed substantially between p8 and p11, with mean coverages of 1187.8× and 83.8×, respectively. Although the lower depth in p11 reduces sensitivity to detect low-frequency variants, both datasets support the same consensus-level structural configuration. Approximately 550 and 96 reads spanning the deletion junction were identified in p8 and p11, respectively, and no reads supporting the corresponding parental configuration were detected. A comparison of the 69 viral CDSs shared by the parental LA, p8, and p11 genomes, excluding ambiguous positions, found no unambiguous SNPs or small indels in p8 relative to LA and one nonsynonymous substitution in p11, in UL28 (L288R). An additional difference in UL36 was detected between p8 and p11, but its ancestral state could not be determined due to unresolved homologous positions. These results show that the consensus genomes were highly conserved, apart from the engineered change and a 3704 nt deletion. Low-frequency variants, especially in the lower-depth p11 dataset, cannot be ruled out. The absence of wild-type reads in the deeply sequenced p8 population, which predates p11, suggests that the wild-type subpopulation later emerging is unlikely. Replacing the deleted region would require reacquisition of the missing sequence rather than reversion, possibly via recombination with a wild-type genome, similar to methods used to reintroduce deleted regions in BoAHV-1 mutants [18].

The loss of US1.67, US2, US3, and part of US4 highlights that additional genomic alterations may occur during the generation or subsequent propagation of recombinant herpesvirus vaccines, even when the intention is to make a single, specific modification. Deletions affecting the US1.67/US2 region of BoAHV-1 have been previously reported [7]. The novelty of the present study lies in the identification and precise mapping of a larger deletion, measuring 3704 nt, which also encompasses the US3 gene and the 5′ portion of the US4 gene in this recombinant vaccine strain previously evaluated in cattle. Previous molecular characterization of BoAHV-1ΔgEβgal by restriction fragment length polymorphism analysis with the *Hind*III enzyme revealed a pattern differing from that expected for the vaccine LA strain, suggesting genomic changes beyond the engineered locus [19]. These observations prompted further genome-wide characterization of the vaccine strain, which enabled the additional structural alteration to be localized and defined at the nucleotide level. The mechanism underlying this unintended deletion remains unclear. However, its location within the US region, near repeated sequences involved in herpesvirus genome architecture, suggests that local recombination may have contributed to its emergence [20,21,22]. Analysis of the sequences flanking the deletion junction revealed no substantial microhomology or direct repeats between the joined ends and no detectable similarity to vector-derived sequences. However, approximately 182 nt adjacent to one breakpoint lie within the IRS and therefore have an inverted counterpart in the TRS. Inverted-repeat regions are recombination-prone components of alphaherpesvirus genomes, and spontaneous BoAHV-1 rearrangements involving duplication/inversion of US sequences together with large deletions have previously been described even in the absence of obvious sequence similarity at the recombination junctions [23]. Thus, the repeat context surrounding the present breakpoint could have facilitated aberrant or ectopic recombination, or another replication-associated rearrangement [24]. However, the molecular mechanism responsible for the deletion cannot be established from the available data.

Functionally, the deleted genes encode proteins involved in key aspects of viral replication and host interaction. US3, a conserved serine/threonine kinase, has been shown to play a central role in cytoskeletal reorganization and in inhibiting apoptosis in BoAHV-1-infected cells, contributing to efficient viral replication and spread [25,26,27,28]. In contrast, US2 is a tegument-associated protein proposed to participate in membrane-associated processes in alphaherpesviruses, although its precise function remains less well defined. In addition, US4 (gG) acts as a chemokine-binding protein that modulates host immune responses and interferes with leukocyte recruitment [10,29]. These functions provide biological context and plausible hypotheses for the consequences of the deletion, but none of these mechanisms were directly examined in the present study.

In the single experimental run available for analysis, BoAHV-1ΔgEβgal p11 showed a lower mean plaque area than the parental LA strain. Previous phenotypic characterization in MDBK cells also reported a marked reduction in plaque size for BoAHV-1ΔgEβgal compared with its parental LA strain and with another BoAHV-1 strain carrying a gE deletion [19]. These observations provide phenotypic context for the recombinant strain; however, the coexistence of the 3704 nt deletion and the reduced plaque-area phenotype does not establish a causal relationship. No isogenic ΔgE/βgal virus lacking the additional 3704 nt deletion or a genetically repaired derivative was available. Therefore, the current plaque dataset describes the phenotype of the complete p11 recombinant virus under the experimental conditions tested and does not establish the functional contribution of the additional deletion. Likewise, the previously published cattle study describes the biological behavior of the complete p11 recombinant strain carrying this genomic configuration but does not establish the contribution of the 3704 nt deletion to in vivo attenuation. However, other previous studies have shown that alterations in genes associated with virulence, particularly those encoding tegument proteins and envelope glycoproteins, result in attenuated phenotypes in alpha-herpesviruses [8,9]. In this context, the deletion identified here involves genes that have also been targeted in experimental strategies aimed at improving vaccine safety, although its functional consequences were not directly assessed in the present study.

A key implication of this study is that targeted genetic engineering strategies, including those used to generate marker vaccines that allow differentiation of infected from vaccinated animals (DIVA), may be accompanied by additional, previously unrecognized genomic rearrangements. Similar events have been documented in herpesviruses and other large DNA viruses, where homologous recombination and replication-associated processes contribute to structural genome variability [22,24,30,31].

Because assays focused on the intended modification may miss genomic alterations outside the engineered locus, genome-wide sequencing can provide a complementary, non-targeted view of recombinant viral genomes. This approach does not replace targeted PCR, Sanger sequencing, restriction analysis, or protein-based assays, which remain appropriate when specific genomic regions or products are under investigation. Rather, high-throughput sequencing can extend targeted molecular characterization by identifying unexpected structural variants elsewhere in the genome [32,33,34].

This study has several limitations. First, plaque-area measurements are based on a single experimental run in one primary bovine cell system and therefore provide descriptive rather than independently replicated phenotypic evidence. Second, short-read sequencing mainly characterized genome architecture and major structural variation, without systematically investigating viral population diversity or low-frequency variants. Third, growth kinetics and complementary assays capable of distinguishing effects on viral replication, particle production, cell-to-cell spread, and cytotoxicity were not conducted. Finally, in the absence of an isogenic recombinant lacking the additional deletion or a repaired derivative, the contribution of the 3704 nt deletion to the phenotype or previously reported in vivo attenuation cannot be determined.

Taken together, this study provides a retrospective genome-wide characterization of a previously evaluated recombinant BoAHV-1 vaccine strain and identifies an unintended 3704 nt multi-gene deletion present in both p8 and p11. In the single-run plaque assay, p11 showed a lower mean plaque area than the parental LA strain; this observation is descriptive and cannot be specifically attributed to the additional deletion. These findings illustrate how genome-wide sequencing can complement targeted molecular methods by identifying unexpected structural variation outside the intended engineered locus and provide a basis for future studies addressing its functional consequences.

**Table 1:** Primers used for PCR amplification of selected regions of the BoAHV-1 genome.

Name	Sequence	Gene	Amplicon Size	Source
**US2F**	TCCGTCGTAAAGCTGACGAAA	*US2* Virion protein	400 bp	This study
**US2R**	GCTATCCTTTAAAGGCCGCAC			
**US3F**	TACAAATCGGCGGCGCCAAA	*US3* serine/threonine protein kinase	700 bp	Maidana et al., 2020 [35]
**US3R**	TTGTTGACGGCCAAGTATAA			
**US8F**	AGGAGACGCAGTTGGCGCTGAC	*US8* envelope glycoprotein E *gE*	281 bp	Schynts et al., 1999 [36]
**US8R**	CAGGAAGTACCAGTCGATGCTG			
**UL44F**	TGTGACTTGGTGCCCATGTCGC	envelope glycoprotein C *gC*	390 bp	Schynts et al., 1999 [36]
**UL44R**	GAGCAAAGCCCCGCCAAGGAG			
**β-galactosidase**	TTGGCGTAAGTGAAGCGACCC	*Lac z + US9*	1596 bp	This study
**FD-R**	GGTACCGAACTGGCACGTAGC			

Primer pairs target genes within the US region (US2, US3, and US8/gE), the UL region (UL44/gC), and the β-galactosidase insertion site. Amplicon sizes are indicated in base pairs (bp). Primer sequences are shown in the 5′-3′ orientation. Sources correspond to previously published primers or those designed in this study.

## Data Availability

Raw Illumina sequencing reads generated in this study have been deposited in the NCBI Sequence Read Archive (SRA) under BioProject accession PRJNA1523938, with BioSample accessions SAMN62939640, SAMN62939641, and SAMN62939642 corresponding to the LA parental strain and recombinant passages p8 and p11, respectively. Annotated consensus genome sequences have been deposited in GenBank under accession numbers PZ943071, PZ943072, and PZ943073, corresponding to the LA parental strain, p8, and p11, respectively.

## Supplementary Materials

The following supporting information can be downloaded at: https://www.mdpi.com/article/10.3390/microorganisms14102396/s1, Table S1. Individual plaque-area measurements for the parental LA strain and BoAHV-1ΔgEβgal p11; Table S2. Distribution of ambiguous nucleotide positions in the BoAHV-1 parental LA, p8, and p11 genome sequences. Ambiguous nucleotide positions were identified from the GenBank-formatted genome sequences and mapped relative to annotated coding sequences (CDSs). For each genome, the table reports genome length, the total number and percentage of undetermined nucleotides (N), their distribution within and outside CDSs, and the number of CDSs affected. For each affected CDS, the table provides the number and proportion of N positions, the number of codons containing at least one N, and the number of indeterminate amino acid residues (X) in the corresponding GenBank translation. Contiguous N positions are also reported as individual blocks with their genomic coordinates and overlap with annotated CDSs. Non-N IUPAC ambiguity codes are also listed. Positions containing ambiguous nucleotides were considered indeterminate and were not classified as synonymous or nonsynonymous substitutions. When CDSs overlap, genome-level counts represent unique nucleotide positions and therefore do not double-count ambiguous positions shared by overlapping CDSs; Table S3. Codon changes between the three draft genomes.
