# Supplementary material for: Genome-Wide Characterization of a Recombinant BoAHV-1 ΔgE/β-gal Vaccine Strain Reveals an Unintended Multi-Gene Deletion
Source: Microorganisms. 2026 Oct 20;14(10):2396. doi: 10.3390/microorganisms14102396 (PMC13707835; doi:10.3390/microorganisms14102396)
Supplement: Supplementary file 1 [file microorganisms-14-02396-s001.zip › microorganisms-4523156-supplementary.pdf]

Supplementary Table S1. Individual plaque-area measurements for the parental LA strain and BoAHV-1ΔgEβgal p11.

LA parental strain	***BoAHV-1ΔgEβgal p11***
8121	2543
9087	2908
7896	2467
7986	2543
8665	2450
8351	2568
8297	2562
7859	2952
8641	2557
7793	2581
9010	2555
7868	
7970	
7947	
9190	
9033	

Supplementary Table S2. Distribution of ambiguous nucleotide positions in the BoAHV-1 parental LA, p8, and p11 genome sequences. Ambiguous nucleotide positions were identified from the GenBank-formatted genome sequences and mapped relative to annotated coding sequences (CDSs). For each genome, the table reports genome length, the total number and percentage of undetermined nucleotides (N), their distribution within and outside CDSs, and the number of CDSs affected. For each affected CDS, the table provides the number and proportion of N positions, the number of codons containing at least one N, and the number of indeterminate amino acid residues (X) in the corresponding GenBank translation. Contiguous N positions are also reported as individual blocks with their genomic coordinates and overlap with annotated CDSs. Non-N IUPAC ambiguity codes are also listed. Positions containing ambiguous nucleotides were considered indeterminate and were not classified as synonymous or nonsynonymous substitutions. When CDSs overlap, genome-level counts represent unique nucleotide positions and therefore do not double-count ambiguous positions shared by overlapping CDSs.

Genome	CDS/gene	Location	CDS length (nt)	N in CDS	N (% CDS)	Codons containing N	X in submitted translation	Product
BoHV1_LA_seed	UL43	[18410:19547](-)	1137	43	3,782	16	14	envelope protein UL43
BoHV1_LA_seed	UL36	[30941:40739](+)	9798	227	2,317	77	77	large tegument protein
BoHV1_LA_seed	UL28	[53149:55630](+)	2481	2	0,081	2	1	DNA packaging terminase subunit 2
BoHV1_LA_seed	UL27	[55485:58287](+)	2802	9	0,321	4	4	envelope glycoprotein B
BoHV1_LA_seed	UL17	[76449:78558](+)	2109	39	1,849	14	13	DNA packaging tegument protein 17
BoHV1_LA_seed	BICP4	[103799:107879](-)	4080	700	17,157	238	236	transcriptional regulator ICP4
BoHV1_LA_seed	BICP22	[112731:113634](+)	903	10	1,107	4	3	regulatory protein BICP22
BoHV1_LA_seed	BICP22	[124187:125090](-)	903	10	1,107	4	3	regulatory protein BICP22

BoHV1_LA_seed	BICP4	[129942:134022](+)	4080	700	17,157	238	236	transcriptional regulator ICP4
BoHV1_DgE_bgal_p8	UL36	[30868:40666](+)	9798	69	0,704	25	24	large tegument protein
BoHV1_DgE_bgal_p8	UL28	[53076:55557](+)	2481	13	0,524	5	4	DNA packaging terminase subunit 2
BoHV1_DgE_bgal_p8	BICP0	[100738:102769](-)	2031	19	0,935	7	7	ubiquitin E3 ligase BICP0
BoHV1_DgE_bgal_p8	LR-OF1	[100949:101963](+)	1014	19	1,874	7	7	product of latency-related protein
BoHV1_DgE_bgal_p11	UL43	[18410:19547](-)	1137	75	6,596	25	25	envelope protein UL43
BoHV1_DgE_bgal_p11	UL36	[30941:40739](+)	9798	244	2,490	84	82	large tegument protein
BoHV1_DgE_bgal_p11	UL32	[42518:44324](+)	1806	25	1,384	9	8	DNA packaging protein UL32
BoHV1_DgE_bgal_p11	UL28	[53149:55630](+)	2481	24	0,967	10	8	DNA packaging terminase subunit 2
BoHV1_DgE_bgal_p11	UL27	[55485:58287](+)	2802	58	2,070	20	19	envelope glycoprotein B
BoHV1_DgE_bgal_p11	UL17	[76449:78558](+)	2109	13	0,616	6	5	DNA packaging tegument protein 17
BoHV1_DgE_bgal_p11	BICP4	[103800:107880](-)	4080	928	22,745	315	314	transcriptional regulator ICP4
BoHV1_DgE_bgal_p11	BICP22	[112734:113637](+)	903	27	2,990	9	9	regulatory protein BICP22

BoHV1_DgE_bgal_p11	BICP22	[123329:124232](-)	903	58	6,423	20	20	regulatory protein BICP22
BoHV1_DgE_bgal_p11	BICP4	[129084:133164](+)	4080	848	20,784	287	287	transcriptional regulator ICP4

Supplementary Table S3. Codon changes between the three draft genomes.

Comparison	Gene	CDS position	Nucleotide change	Codon change	Amino-acid change	Classification		
LA vs p8	—	—	None	—	—	No unambiguous coding SNPs		
LA vs p11	UL28	863	T→G	CTG→CGG	L288R	Nonsynonymous		
p8 vs p11*	UL36	1265	T→G	CTG→CGG	L422R	Nonsynonymous		

* The corresponding parental LA position is unresolved (N); therefore, this difference was not classified as a parental-to-vaccine variant.
